# Longitudinal analysis of T1w/T2w ratio in patients with multiple sclerosis from first clinical presentation

**DOI:** 10.1177/13524585211003479

**Published:** 2021-04-15

**Authors:** Graham Cooper, Claudia Chien, Hanna Zimmermann, Judith Bellmann-Strobl, Klemens Ruprecht, Joseph Kuchling, Susanna Asseyer, Alexander U Brandt, Michael Scheel, Carsten Finke, Friedemann Paul

**Affiliations:** Experimental and Clinical Research Center, Max Delbrueck Center for Molecular Medicine and Charité—Universitätsmedizin Berlin, corporate member of Freie Universität Berlin, Humboldt-Universität zu Berlin and Berlin Institute of Health, Berlin, Germany/NeuroCure Clinical Research Center, Charité—Universitätsmedizin Berlin, corporate member of Freie Universität Berlin, Humboldt-Universität zu Berlin and Berlin Institute of Health, Berlin, Germany/ Department of Experimental Neurology and Center for Stroke Research, Berlin, Charité—Universitätsmedizin Berlin, Berlin, Germany/ Einstein Center for Neurosciences, Berlin, Germany; Experimental and Clinical Research Center, Max Delbrueck Center for Molecular Medicine and Charité—Universitätsmedizin Berlin, corporate member of Freie Universität Berlin, Humboldt-Universität zu Berlin and Berlin Institute of Health, Berlin, Germany/NeuroCure Clinical Research Center, Charité—Universitätsmedizin Berlin, corporate member of Freie Universität Berlin, Humboldt-Universität zu Berlin and Berlin Institute of Health, Berlin, Germany/ Department for Psychiatry and Psychotherapy—Charité Universitätsmedizin Berlin, corporate member of Freie Universität Berlin, Humboldt-Universität zu Berlin, and Berlin Institute of Health, Berlin, Germany; Experimental and Clinical Research Center, Max Delbrueck Center for Molecular Medicine and Charité—Universitätsmedizin Berlin, corporate member of Freie Universität Berlin, Humboldt-Universität zu Berlin and Berlin Institute of Health, Berlin, Germany/ NeuroCure Clinical Research Center, Charité—Universitätsmedizin Berlin, corporate member of Freie Universität Berlin, Humboldt-Universität zu Berlin and Berlin Institute of Health, Berlin, Germany; Experimental and Clinical Research Center, Max Delbrueck Center for Molecular Medicine and Charité—Universitätsmedizin Berlin, corporate member of Freie Universität Berlin, Humboldt-Universität zu Berlin and Berlin Institute of Health, Berlin, Germany/ NeuroCure Clinical Research Center, Charité—Universitätsmedizin Berlin, corporate member of Freie Universität Berlin, Humboldt-Universität zu Berlin and Berlin Institute of Health, Berlin, Germany; Department of Neurology, Charité—Universitätsmedizin Berlin, corporate member of Freie Universität Berlin, Humboldt-Universität zu Berlin and Berlin Institute of Health, Berlin, Germany; NeuroCure Clinical Research Center, Charité—Universitätsmedizin Berlin, corporate member of Freie Universität Berlin, Humboldt-Universität zu Berlin and Berlin Institute of Health, Berlin, Germany/Department of Neurology, Charité—Universitätsmedizin Berlin, corporate member of Freie Universität Berlin, Humboldt-Universität zu Berlin and Berlin Institute of Health, Berlin, Germany; NeuroCure Clinical Research Center, Charité—Universitätsmedizin Berlin, corporate member of Freie Universität Berlin, Humboldt-Universität zu Berlin and Berlin Institute of Health, Berlin, Germany/Department of Neurology, Charité—Universitätsmedizin Berlin, corporate member of Freie Universität Berlin, Humboldt-Universität zu Berlin and Berlin Institute of Health, Berlin, Germany; NeuroCure Clinical Research Center, Charité—Universitätsmedizin Berlin, corporate member of Freie Universität Berlin, Humboldt-Universität zu Berlin and Berlin Institute of Health, Berlin, Germany/Department of Neurology, University of California, Irvine, California, USA; NeuroCure Clinical Research Center, Charité—Universitätsmedizin Berlin, corporate member of Freie Universität Berlin, Humboldt-Universität zu Berlin and Berlin Institute of Health, Berlin, Germany/Department of Neuroradiology, Charité—Universitätsmedizin Berlin, corporate member of Freie Universität Berlin, Humboldt-Universität zu Berlin and Berlin Institute of Health, Berlin, Germany; Einstein Center for Neurosciences, Berlin, Germany/Department of Neurology, Charité—Universitätsmedizin Berlin, corporate member of Freie Universität Berlin, Humboldt-Universität zu Berlin and Berlin Institute of Health, Berlin, Germany/Berlin School of Mind and Brain, Humboldt-Universität zu Berlin, Berlin, Germany; Experimental and Clinical Research Center, Max Delbrueck Center for Molecular Medicine and Charité—Universitätsmedizin Berlin, corporate member of Freie Universität Berlin, Humboldt-Universität zu Berlin and Berlin Institute of Health, Berlin, Germany/ NeuroCure Clinical Research Center, Charité—Universitätsmedizin Berlin, corporate member of Freie Universität Berlin, Humboldt-Universität zu Berlin and Berlin Institute of Health, Berlin, Germany/Einstein Center for Neurosciences, Berlin, Germany/Department of Neurology, Charité—Universitätsmedizin Berlin, corporate member of Freie Universität Berlin, Humboldt-Universität zu Berlin and Berlin Institute of Health, Berlin, Germany

**Keywords:** T1w/T2w ratio, multiple sclerosis, normal appearing white matter damage, cortical thickness, longitudinal analysis, NEDA-3

## Abstract

**Background:**

Cross-sectional studies suggest normal appearing white matter (NAWM) integrity loss may lead to cortical atrophy in late-stage relapsing-remitting multiple sclerosis (MS).

**Objective:**

To investigate the relationship between NAWM integrity and cortical thickness from first clinical presentation longitudinally.

**Methods:**

NAWM integrity and cortical thickness were assessed with 3T magnetic resonance imaging (MRI) in 102 patients with clinically isolated syndrome or early MS (33.2 (20.1–60.1) years old, 68% female) from first clinical presentation over 2.8 ± 1.6 years. Fifty healthy controls (HCs) matched for age and sex were included. NAWM integrity was evaluated using the standardized T1w/T2w ratio (sT1w/T2w). The association between sT1w/T2w and cortical thickness was assessed using linear mixed models. The effect of disease activity was investigated using the No Evidence of Disease Activity (NEDA-3) criteria.

**Results:**

At baseline, sT1w/T2w (*p* = 0.152) and cortical thickness (*p* = 0.489) did not differ from HCs. Longitudinally, decreasing sT1w/T2w was associated with cortical thickness and increasing lesion burden (marginal *R*^2^ = 0.061). The association was modulated by failing NEDA-3 (marginal *R*^2^ = 0.097).

**Conclusion:**

sT1w/T2w may be a useful MRI biomarker for early MS, detecting relevant NAWM damage over time using conventional MRI scans, although with less sensitivity compared to quantitative measures.

## Introduction

Magnetic resonance imaging (MRI) is essential for multiple sclerosis (MS) diagnosis, monitoring, and treatment evaluation, enabling not only the detection of lesions in the white and gray matter^1^ but also cortical atrophy^2^ and extralesional white matter damage, referred to as normal-appearing white matter (NAWM).^3^

Diffusion tensor imaging (DTI) findings show that NAWM damage is associated with reduced cortical thickness, an indicator of cortical atrophy, in relapsing-remitting MS (RRMS) patients with a disease duration of 7–9 years.^4,5^ These findings indicate that neurodegenerative cortical atrophy in RRMS may be a retrograde process resulting, at least in part, from NAWM pathology. This is in contrast to the progressive stage, where studies show that atrophy and NAWM pathology correlate less and may have developed into distinct processes.^4,6–8^ However, previous studies were limited by their cross-sectional design, making it difficult to draw conclusions about the temporal relationship between the two processes. Furthermore, the influence of accumulating disease activity (e.g. new lesions, relapses) on NAWM integrity has not yet been investigated.

Although the clinical relevance of NAWM is well-established, particularly in relation to cognitive impairment,^3,9^ NAWM integrity measurement is not yet included in the clinical routine. This may be because state-of-the-art techniques for measuring NAWM integrity, such as DTI,^5,6^ require additional scanning time and post-processing expertise that cannot be readily incorporated into the clinic.^10^ Originally developed as a marker of myelination, the T1/T2-weighted ratio (T1w/T2w) has been shown to be sensitive to NAWM pathology in RRMS from 2 years disease duration.^11,12^ T1w/T2w is particularly promising as it is based on scans acquired as part of the clinical routine and the necessary post-processing methods are fast and easily implemented. Recently, standardizing T1w/T2w (sT1w/T2w) using the combined image method, originally developed for contrast enhancement,^13^ has been shown to increase sensitivity to NAWM damage in MS compared to the conventional T1w/T2w^14^ and T1w/T2w has been shown to correlate with other quantitative measures of NAWM damage in MS.^15^ However, longitudinal investigations of NAWM integrity in patients with MS using T1w/T2w, which are critical to evaluate the clinical utility of this measure, are lacking.

Here, we investigate the association between NAWM sT1w/T2w and cortical thickness in early MS/clinically isolated syndrome (CIS) patients from first clinical presentation over a mean of 2.8 ± 1.6 years. First, we investigate baseline differences between early MS/CIS and healthy controls to determine the sensitivity of sT1w/T2w to NAWM damage in early disease stage. We also investigate cross-sectional differences between early MS/CIS and late RRMS to confirm the disease effect and directionality (reduced sT1w/T2w in MS). We then test various linear mixed models to investigate whether NAWM sT1w/T2w is associated with cortical thickness and lesions over time and, additionally, whether these changes are modulated by disease activity measured by the “No Evidence of Disease Activity” (NEDA-3^16^) criteria.

## Materials and methods

### Study population

Data of 173 patients from an ongoing longitudinal prospective observational study at the NeuroCure Clinical Research Center (NCRC), Charité-Universitätsmedizin Berlin (clinical trial number NCT01371071), were screened for inclusion in this study. Patients were recruited between March 2011 and September 2019. We included patients that, at baseline, were at least 18 years old and diagnosed with CIS or early RRMS according to the 2017 McDonald criteria.^1^ Exclusion criteria were less than two follow-up visits (*n* = 47), a diagnosis other than CIS or RRMS (*n* = 18), or a disease duration longer than 13 months at baseline (*n* = 6). No restrictions based on disease-modifying therapy were applied. No patients were on maintenance corticosteroid therapy at inclusion and the temporal distance between previous relapse and high dose methylprednisolone was at least 30 days. Given that sT1w/T2w is a novel method, cross-sectional data of 114 patients from another ongoing prospective study at NCRC were also screened for inclusion in this study, to confirm previous findings of NAWM damage in RRMS.^14^ Patients were recruited between October 2015 and November 2018. We included patients who, at baseline, were at least 18 years old, diagnosed with RRMS according to the 2017 McDonald criteria, and had a disease duration longer than 10 years.

A cross-sectional data set of 50 healthy controls matched for age at baseline (±6 months) and sex was recruited between June 2015 and March 2019 in order to compare MRI parameters with the patient cohort at baseline. Neither healthy control nor late RRMS data were used in the longitudinal analysis.

This study was approved by the local ethics committee (Ethikkommission der Charité-Universitätsmedizin Berlin; EA1/182/10 and EA1/163/12) and conducted in accordance with the current applicable version of the Declaration of Helsinki and German law. All participants provided written informed consent. There is no overlap of patient data between the present study and previous publication.^14^

### MRI acquisition

MRI acquisition was performed on a 3 Tesla MRI (Tim Trio, Siemens Medical Systems, Erlangen, Germany) at the Berlin Center of Advanced Neuroimaging (BCAN; www.berlin-can.de). The MRI protocol included a 3D T1w magnetization–prepared rapid acquisition gradient echo (repetition time (TR) = 1900 ms, echo time (TE) = 2.55 ms, inversion time (TI) = 900 ms, 1 mm isotropic resolution), a 3D T2w sampling perfection with application-optimized contrasts using flip angle evolution (TR = 5000 ms, TE = 502 ms, 1 mm isotropic resolution), and a 3D T2w fluid-attenuated inversion recovery (FLAIR, TR = 6000 ms, TE = 388 ms, TI = 2100 ms, 1 mm isotropic resolution) sequence.

### MRI analysis

T1w, T2w, and FLAIR were bias-field corrected with non-parametric, non-uniform intensity normalization.^16^ At baseline, all images were linearly co-registered to MNI space with a spline interpolation using FMRIB’s Linear Image Registration Tool from the FMRIB Software Library (FSL) version 5.0.9.^17^ Follow-up images were linearly co-registered to MNI space and then to the baseline FLAIR image for each patient. The transformation matrices were combined so that only one interpolation was necessary.

Lesion segmentation was conducted semi-automatically with FLAIR images using the lesion prediction algorithm from the Lesion Segmentation Toolbox version 2.0.15 (www.statistical-modeling.de/lst.html) for SPM12 version 7219 in MATLAB 2016b and manually corrected using ITK-SNAP (www.itksnap.org). New lesions were manually identified and labeled by trained raters under supervision of a board-certified radiologist. Lesion volume (mL) and the number of new lesions at each follow-up visit were calculated using the FSL cluster algorithm.

Generation of a gray matter, white matter, and cerebrospinal fluid mask, and calculation of mean cortical thickness were performed using lesion-filled T1w images with the Computational Anatomy Toolbox (CAT12) version 11.09 (http://www.neuro.uni-jena.de/cat12/CAT12-Manual.pdf) for SPM12. Normal-appearing tissue masks for gray and white matter were created using FSL by subtracting the lesion mask from the respective tissue mask.

To generate whole brain sT1w/T2w images, a scaling factor was calculated by dividing the median normal-appearing gray matter intensity of the T1w image by the median normal-appearing gray matter intensity of the T2w image. The T2w image was then multiplied by the scaling factor to create a scaled T2w image. sT1w/T2w was then calculated using the following equation^13^

$$sT1w/T2w=\frac{T1w-scaled\;T2w}{T1w+scaled\;T2w}$$

The median NAWM sT1w/T2w was extracted for each subject. The conventional (non-standardized) T1w/T2w was also calculated as previously described.^14^ The same sequences described above were used for the sensitivity analyses.

### Clinical assessment

At each visit, patients received a neurological examination including the Expanded Disability Status Scale (EDSS) score from trained physicians supervised by a board-certified neurologist.

The NEDA-3 criteria were used as the primary measure of disease progression.^18^ NEDA-3 criteria are met if patients (1) did not have a relapse, (2) have no new or enlarging T2w or T1w gadolinium-enhancing lesions, and (3) their EDSS score is stable since their last visit. An increase in the EDSS was defined as an increase by 1 (1.5 if previous EDSS < 1 and 0.5 if previous EDSS > 5.5). Gadolinium-enhanced T1w scans were not acquired at follow-up visits so only the absence of new T2w-FLAIR lesions was used for (2). Given this limitation, we refer to this measure as the modified NEDA-3 in this paper. Patients were assessed for modified NEDA-3 at each follow-up visit. Failure to meet any of the three criteria was counted as a failure to meet modified NEDA-3.

### Statistical analysis

Group-level comparisons of categorical variables were conducted using the Chi-squared test. Student’s *t*-test or Wilcoxon test was used for continuous variables, as appropriate. Correlations were investigated using Pearson’s test.

In order to assess whether sT1w/T2w changed meaningfully over the disease course, we calculated linear mixed models (random intercept models) for NAWM sT1w/T2w values using the lmerTest and lme4 packages. In all models, the repeated measurements of sT1w/T2w were level-one units nested in the different patients, who represented level-two units. Follow-up time and age at baseline were included in all models as fixed effects to investigate the association with time and control for age effects, respectively. To investigate the association with cortical thickness and lesion volume over time, three models were calculated and compared: (1) including the interaction between lesion volume and follow-up time as a fixed effect, (2) including the interaction between cortical thickness and follow-up time as a fixed effect, and (3) including the interaction between follow-up time and both imaging parameters as fixed effects. Once the association with imaging parameters was determined, we additionally investigated whether and how disease activity (failing modified NEDA-3) influenced the model. To do this we added modified NEDA-3 to the model in the following ways: (1) including NEDA-3 as a fixed effect, (2) including the interaction between modified NEDA-3 and follow-up time as a fixed effect, and (3) including modified NEDA-3 as a random slope. Models were compared using analysis of variance (ANOVA). The formulae for each linear mixed model are presented in the Supplementary Data.

Statistical analysis was conducted using R version 3.5.1.^19^ Statistical significance was set at *p* < 0.05.

## Results

### Group-level comparisons

**Figure 1.**
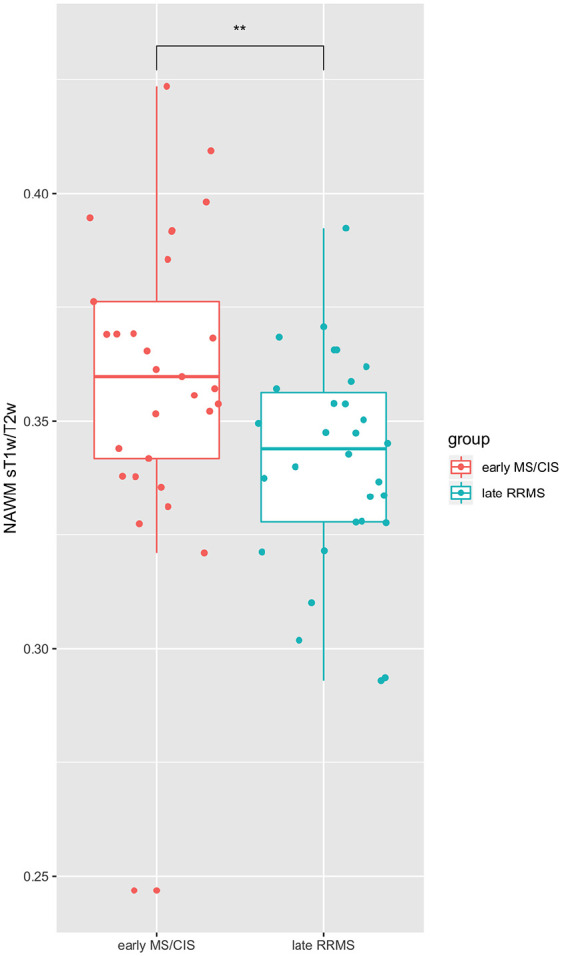
Comparison of baseline NAWM sT1w/T2w between early MS/CIS patients and late RRMS patients. **p < 0.05.

Table 1 shows the demographic, MRI, and clinical characteristics at baseline for early MS/CIS patients and healthy controls. Disease duration at baseline reflects the time between first symptom and baseline MRI acquisition. At baseline, NAWM sT1w/T2w and cortical thickness did not differ between early MS/CIS patients and controls. Table 2 shows the same characteristics for the late RRMS cohort compared with a subgroup of early MS/CIS patients matched for age and sex. The late RRMS cohort had significantly lower NAWM sT1w/T2w and cortical thickness compared to the early MS/CIS cohort (Table 2, Figure 1). The conventional T1w/T2w and sT1w/T2w were significantly correlated (Pearson’s *R* = 0.41, *p* < 0.001).

**Table 1.** Demographic, imaging, and clinical characteristics of early MS and healthy controls.

	HC	Early MS/CIS	Test statistic	Significance (*p*-value)
*N*	50	102		
Age (median, range) (years)	31.8 (19.5–69.0)	33.2 (20.1–60.1)	*t* = 1.65	0.103
Sex = M (%)	16 (32%)	34 (35.1%)	χ^2^ = 152	0.439
Disease duration (median, range) (months)		4.2 (0–13)		
Diagnosis at baseline = CIS (%)		28 (27.5%)		
Diagnosis at last visit = CIS (%)		16 (22.9%)		
Follow-up duration (median, range) (months)		33.6 (4.3–70.5)		
EDSS (median, range)		1.5 (0–5)		
T2 lesion volume (mL) (mean)		2.41 ± 3.43		
Cortical thickness (mm) (mean)	2.62 ± 0.14	2.63 ± 0.11	*t* = −0.70	0.489
NAWM sT1w/T2w (mean)	0.37 ± 0.03	0.37 ± 0.03	*t* = 1.44	0.152
Fail NEDA-3 during follow-up (%) Increased EDSS Relapse New T2 lesions		86 (84.3%) 34 (39.5%) 38 (44.2%) 51 (59.3%)		
Therapy at baseline (%) Interferon beta Glatiramer acetate Fumarate Immunoglobins Other		34 (33.3%) 10 (29.4%) 16 (47.1%) 6 (17.6%) 1 (2.9%) 1 (2.9%)		
Therapy at last visit (%) Interferon beta Glatiramer acetate Fumarate Immunoglobins Natalizumab Cyclophosphamide Other		27 (26.5%) 8 (29.6%) 6 (22.2%) 6 (22.2%) 1 (3.7%) 2 (7.4%) 1 (3.7%) 2 (7.4%)		

HC: healthy control; MS: multiple sclerosis; CIS: clinically isolated syndrome; NAWM: normal-appearing white matter; NEDA-3: No Evidence of Disease Activity.

**Table 2.** Demographic, imaging, and clinical characteristics of late RRMS and early MS/CIS patients.

	Early MS/CIS	Late RRMS	Test statistic	Significance (*p*-value)
*N*	30	30		
Age (median, range) (years)	41.15 (37.1–56.2)	45.60 (32.0–60.2)	*t* = −1.752	0.086
Sex = M (%)	8 (26.7%)	12 (40%)	χ^2^ = 0.68	0.4113
Disease duration (median, range) (months)	5(1–10)	186 (124–444)	*W* = 0	<0.0001
EDSS (median, range)	1.75 (0–3)	2.00 (0–4.5)	χ^2^ = 10.80	0.214
T2 lesion volume (mL) (mean)	1.96 ± 3.13	10.46 ± 10.97	*W* = 154	<0.0001
Cortical thickness (mm) (mean)	2.58 ± 0.09	2.51 ± 0.09	*t* = 2.85	0.006
NAWM sT1w/T2w (mean)	0.36 ± 0.03	0.34 ± 0.02	*t* = 2.45	0.018

RRMS: relapsing-remitting multiple sclerosis; MS: multiple sclerosis; CIS: clinically isolated syndrome; EDSS: Expanded Disability Status Scale; NAWM: normal-appearing white matter.

#### Longitudinal trajectory of imaging parameters

**Figure 2.**
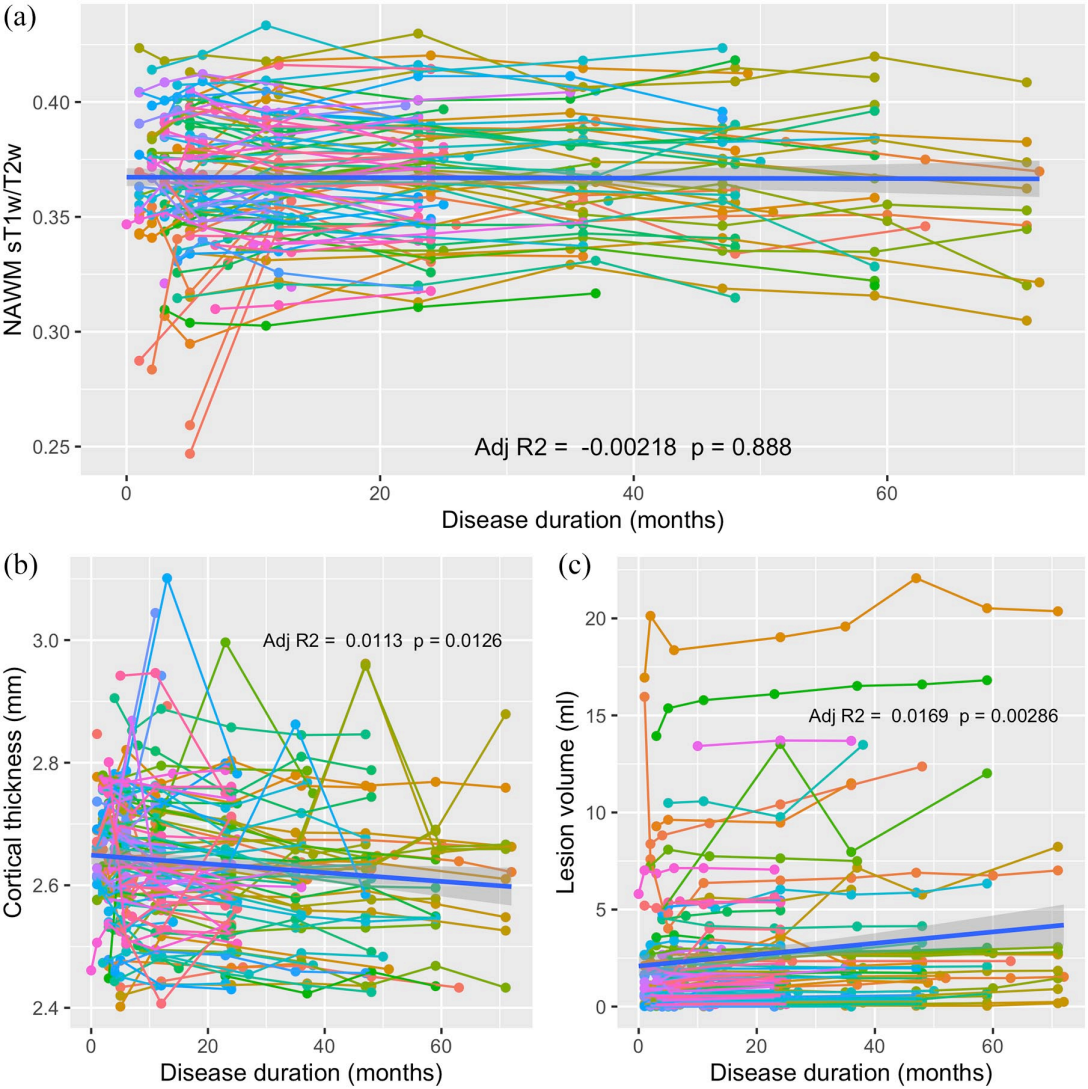
NAWM sT1w/T2w ratio (a), cortical thickness (b), and lesion volume (c) plotted against disease duration (months). Individual colored lines show the trajectory of each parameter for individual patients. The thick blue line shows the regression line for the model parameter ~ disease duration for the whole cohort, including 95% confidence intervals (transparent blue). Adjusted *R*^2^ and *p*-values are shown for this regression.

Figure 2 shows the trajectory of all imaging parameters over time. Cortical thickness significantly decreased and lesion volume significantly increased over time, while no significant change in NAWM sT1w/T2w was detected.

### Individual trajectories of white matter damage: Association with imaging parameters

The association between NAWM sT1w/T2w over time and other imaging parameters over time was assessed by fitting a linear mixed model of NAWM sT1w/T2w values with subject as a random effect and comparing the model after the following conditional likelihood effects interacting with follow-up time were added: (1) lesion volume, (2) cortical thickness, (3) cortical thickness and lesion volume. Model 3 was the best model (*p* = 0.004). Lower NAWM sT1w/T2w was associated with lower cortical thickness and increasing lesion volume (Table 3).

**Table 3.** Comparison of the imaging association models.

	Model 1	Model 2	Model 3
Age at baseline coefficient (95% CI)	−0.0002 ± 0.0003	0.00002 ± 0.0003	−0.00004 ± 0.0003
Follow-up time coefficient (95% CI)	−0.0001 ± 0.0001	0.001 ± 0.001	0.001 ± 0.001
Lesion volume coefficient (95% CI)	−0.009 ± 0.003**		−0.009 ± 0.003**
Cortical thickness coefficient (95% CI)		0.036 ± 0.013**	0.032 ± 0.013**
Interaction between lesion volume and follow-up time coefficient (95% CI)	0.0001 ± 0.00004**		0.0001 ± 0.00004**
Interaction between cortical thickness and follow-up time coefficient (95% CI)		−0.001 ± 0.0003	−0.005 ± 0.0003
Intercept (95% CI)	0.381 ± 0.011**	0.271 ± 0.037**	0.289 ± 0.038**
Number of patients	102	102	102
Number observations	449	449	449
Marginal *R*^2^	0.051	0.013	0.061
Conditional *R*^2^	0.837	0.844	0.839
AIC	−2381	−2384	−2362

CI: confidence interval; AIC: Akaike information criterion.

** *p* < 0.01.

#### Association with NEDA-3

The effect of failing NEDA-3 was explored by adding the following terms to the model with lesion volume and cortical thickness interacting with follow-up time: (1) NEDA-3, (2), NEDA-3 and the interaction between NEDA-3 and follow-up time, and (3) NEDA-3 as a random slope. In all three models, the marginal *R*^2^ increased by ~40% compared to the models without NEDA-3, indicating that NEDA-3 status explained additional variability in NAWM sT1w/T2w (Table 4). The best model of NEDA-3 was model 3 (*p* < 0.001). Therefore, lower NAWM sT1w/T2w was associated with lower cortical thickness and increasing lesion volume, adjusting for age at baseline, and the steepness of the association was higher if patients failed NEDA-3 at each visit.

**Table 4.** Comparison of the three NEDA-3 models.

	Model 1	Model 2	Model 3
Age at baseline coefficient (95% CI)	−0.00001 ± 0.0003	−0.00001 ± 0.0003	−0.00005 ± 0.0003
Follow-up time coefficient (95% CI)	0.001 ± 0.001	0.001 ± 0.001	0.0005 ± 0.001
Lesion volume coefficient (95% CI)	−0.011 ± 0.003**	−0.011 ± 0.003**	−0.011 ± 0.003**
Cortical thickness coefficient (95% CI)	0.031 ± 0.011**	0.031 ± 0.011**	0.029 ± 0.011**
Interaction between lesion volume and follow-up time coefficient (95% CI)	0.0001 ± 0.00004*	0.0001 ± 0.00004*	0.0001 ± 0.00004*
Interaction between cortical thickness and follow-up time coefficient (95% CI)	−0.0003 ± 0.0003	−0.0003 ± 0.0003	−0.0002 ± 0.0003
NEDA-3 coefficient (95% CI)	0.001 ± 0.001	0.001 ± 0.002	
Interaction between NEDA-3 and follow-up time coefficient (95% CI)		0.00001 ± 0.0001	
Intercept (95% CI)	0.296 ± 0.034**	0.296 ± 0.034**	0.302 ± 0.033**
Number of patients	102	102	102
Number observations	352	352	352
Marginal *R*^2^	0.100	0.100	0.097
Conditional *R*^2^	0.906	0.906	0.913
AIC	−1946	−1926	−1958

NEDA-3: No Evidence of Disease Activity; CI: confidence interval; AIC: Akaike information criterion.

* *p* < 0.05; **p < 0.01.

### Sensitivity analyses

Despite not being sensitive to detect cross-sectional differences at first clinical presentation, the longitudinal analysis showed that sT1w/T2w is associated with lesion volume, modified NEDA-3, and cortical thickness, indicating clinical relevance. We therefore first compared lesion volume, cortical thickness, and sT1w/T2w between patients with CIS and RRMS at baseline. No differences in cortical thickness (*t* = 0.885, *p* = 0.380) or sT1w/T2w (*t* = 1.598, *p* = 0.117) were detected, but RRMS patients had significantly higher lesion volumes (*t* = -6.3398, *p* < 0.001). Next, we saw an apparent difference in lesion distribution over time with some patients having a stable lesion volume and others increasing over time (Figure 2(a)). We therefore split patients into two groups based on baseline lesion volume (>2 mL). Patients with a baseline lesion volume >2 mL had significantly reduced sT1w/T2w compared to controls and those with a baseline lesion volume <2 mL (Supplementary Data).

As sT1w/T2w has not previously been investigated longitudinally, we additionally evaluated whether partial volume effects or gray matter pathology had a systematic influence on sT1w/T2w. We first recalculated the sT1w/T2w using an eroded gray and white matter mask to remove any voxels with potential partial volume effects and then repeated the cross-sectional comparison between early MS/CIS and healthy controls as well as the linear mixed model analysis investigating the association with imaging parameters and NEDA-3. The same results were found using the eroded sT1w/T2w (Supplementary Data). In order to evaluate the effect of gray matter pathology, we first compared the median gray matter T1w and T2w intensity values and the scaling factor between early MS/CIS and healthy controls at baseline. No significant differences were found: T1w (266.52 vs 275.46, *p* = 0.175), T2w (73.35 vs 73.59, *p* = 0.983), and scaling factor (3.76 vs 3.84, *p* = 0.477). We also evaluated whether the scaling factor was driven by gray matter pathology over time and found only a small effect of age (Supplementary Data).

Finally, given the presence of potential outliers in sT1w/T2w Figure 2(a) we used Cooks’ distance analysis to detect values that were overly influencing the results of model 3 investigating the association of modified NEDA-3 and imaging parameters on sT1w/T2w over time. Six influential data points were identified, and this model was repeated without these data points. The results remained unchanged (Data not shown).

## Discussion

This study showed that decreased NAWM integrity measured by sT1w/T2w was associated with reduced cortical thickness and an increasing lesion burden during the first years of disease in patients with early MS/CIS. This association was modulated by increased disease activity, measured by modified NEDA-3. The study also confirmed previous findings of reduced sT1w/T2w later in the disease course^14^ although sT1w/T2w was not sensitive to detect NAWM damage at first clinical presentation (baseline) in the whole cohort.

### NAWM damage: An important marker of neurodegeneration in early MS

Cortical atrophy is becoming increasingly recognized as a critical marker of neurodegeneration in MS.^20–22^ However, consistent positive effects of treatment on cortical atrophy are only reported after 1 year of disease,^23^ and at this point, irreversible neurodegeneration is likely to have already occurred. Therefore, techniques sensitive to detect neurodegenerative damage prior to atrophy are needed.^23^ NAWM damage, characterized by demyelination, gliosis, axonal loss, as well as mitochondrial injury and ionic dysregulation,^3,24,25^ can be detected early in the disease course^11,26^ and has been highlighted as one such target.

Further support for the measurement of NAWM integrity in early MS comes from recent evidence suggesting that NAWM damage, as measured by DTI, and cortical atrophy are related processes in early MS. Associations between NAWM diffusivity changes and cortical thinning in connected areas have been reported, particularly in RRMS.^4–6^ However, these studies were conducted at a disease duration (2–7 years) where irreversible neurodegeneration, atrophy, was already apparent. This study builds on these, showing that NAWM integrity is associated with cortical thickness from first clinical presentation, before cortical atrophy can be detected at the group level. Large changes in NAWM integrity during the first 2 years were not detected in this cohort, supporting findings from Rocca et al.,^26^ who did not detect longitudinal changes in the first 2 years of disease course in early MS/CIS patients. In contrast however, our study did not detect cross-sectional differences in NAWM integrity compared to controls using sT1w/T2w, despite detecting NAWM integrity loss in later RRMS and previous work,^14^ as well as in the subgroup of patients with a higher lesion burden. We suggest that sT1w/T2w is not sensitive enough to detect the initial heterogeneous amount of NAWM damage that would have occurred at baseline. An alternative explanation may be that the baseline cohort was much earlier in the disease course compared to previous studies and additional inflammation and edema may have influenced the results. Nonetheless, our findings with sT1w/T2w support previous findings suggesting that NAWM damage is an early neurodegenerative marker antecedent to cortical neurodegeneration that represents a potential outcome measure for early treatment decisions.

### NEDA-3 and NAWM damage

NAWM integrity was associated with failing modified NEDA-3, a common secondary outcome measure in clinical trials thought to be a more holistic marker of disease progression.^27^ Recent evidence showed that NEDA-3 status at 2 years had a high positive predictive value of no disability progression at 7 years,^18^ although contrasting results and worsening cognition and mobility despite meeting NEDA-3 have also been reported.^28,29^ Only two studies have investigated the association between NAWM damage and NEDA status. Cramer et al.^30^ found that increased NAWM permeability at baseline predicted NEDA status at 2 years. Harel et al.^31^ found increased NAWM diffusivity, irrespective of NEDA status. This study found that including modified NEDA-3 as a random slope in the linear mixed model significantly improved model fit and increased effect sizes, suggesting that patients who failed modified NEDA-3 at a given time point had a higher decrease in NAWM integrity associated with cortical thickness and lesion volume. Despite the clinically stable cohort, these results suggest that NAWM integrity, as measured by sT1w/T2w, is a clinically relevant marker from disease onset, prior to irreversible neurodegeneration and also debilitating clinical manifestations.

### sT1w/T2w as a measure of NAWM damage

sT1w/T2w is a relatively novel measure of NAWM integrity damage in MS, standardizing the conventional T1w/T2w using the combined image technique, originally developed for contrast enhancement.^13^ The conventional T1w/T2w has been shown to be sensitive to MS pathology, both in the cortex^32^ and NAWM,^11,12,15^ and is related to reduced EDSS and cognitive impairment.^11,32^ The main benefit of the conventional T1w/T2w compared to other markers of NAWM damage is that it uses routine clinical images and has relatively simple post-processing steps. T1w/T2w is, however, susceptible to technical limitations and therefore calibration and standardization methods have been proposed.^13,33^ sT1w/T2w has been shown to correct for the effect of bias fields, reduce variability, and increase sensitivity to MS damage in NAWM, compared to the conventional T1w/T2w^13,14^ and is easily implemented using standard segmentation and post-processing steps compared to other methods.^33^ While sT1w/T2w cannot be reduced to the conventional T1w/T2w, this study confirms previous findings that both measures are well correlated,^14^ and we propose that the differences in findings come from the standardization method based on gray matter. However, the gray matter standardization may cause sT1w/T2w to be influenced by partial volume effects and gray matter pathology, such as cortical lesions, which were not able to be identified using the available data, resulting in a less valid method of NAWM integrity. We believe that these effects are minimized in the current cohort for the following reasons: (1) we used CAT12 for segmentation, which corrects for partial volume effects;^34^ (2) we reran the analysis using eroded tissue masks and the linear mixed model results remained stable; and (3) we show that, in this cohort, the scaling factor is not influenced by disease factors and that the T1w and T2w gray matter intensities are not sensitive to pathology, confirming previous findings.^14^ In addition, the findings that sT1w/T2w are correlated with lesion volume is consistent with recent publications using the conventional T1w/T2w and T1w/T2w corrected using cerebrospinal fluid,^12,35^ further supporting the similarity of these methods. Nonetheless future studies investigating sT1w/T2w over time in healthy controls as well as studies specifically designed to further investigate the impact of gray matter pathology on the validity of the calibration are necessary to help interpret the results presented here. It should also be noted that, while T1w/T2w is accepted as a marker of microstructural integrity in the whole brain,^11,14,32^ the exact pathological substrate of T1w/T2w has not yet been determined. Combined MRI-histopathology studies to determine the pathological substrate of T1w/T2w in NAWM, similar to those already conducted in the gray matter,^32,36,37^ are lacking and are required to better understand the underlying damage shown by this method.

## Conclusion

In conclusion, NAWM integrity measured by sT1w/T2w is associated with increasing lesion volume, failing modified NEDA-3 criteria, and cortical thickness in the first 2 years of disease after first clinical presentation. Although less sensitive than other established methods, sT1w/T2w represents a promising measure of NAWM integrity for retrospective clinical studies without access to more advanced measures, such as DTI.

## Supplemental Material

sj-pdf-1-msj-10.1177_13524585211003479 – Supplemental material for Longitudinal analysis of T1w/T2w ratio in patients with multiple sclerosis from first clinical presentation Click here for additional data file. Supplemental material, sj-pdf-1-msj-10.1177_13524585211003479 for Longitudinal analysis of T1w/T2w ratio in patients with multiple sclerosis from first clinical presentation by Graham Cooper, Claudia Chien, Hanna Zimmermann, Judith Bellmann-Strobl, Klemens Ruprecht, Joseph Kuchling, Susanna Asseyer, Alexander U Brandt, Michael Scheel, Carsten Finke and Friedemann Paul in Multiple Sclerosis Journal

sj-pdf-2-msj-10.1177_13524585211003479 – Supplemental material for Longitudinal analysis of T1w/T2w ratio in patients with multiple sclerosis from first clinical presentation Click here for additional data file. Supplemental material, sj-pdf-2-msj-10.1177_13524585211003479 for Longitudinal analysis of T1w/T2w ratio in patients with multiple sclerosis from first clinical presentation by Graham Cooper, Claudia Chien, Hanna Zimmermann, Judith Bellmann-Strobl, Klemens Ruprecht, Joseph Kuchling, Susanna Asseyer, Alexander U Brandt, Michael Scheel, Carsten Finke and Friedemann Paul in Multiple Sclerosis Journal

sj-pdf-3-msj-10.1177_13524585211003479 – Supplemental material for Longitudinal analysis of T1w/T2w ratio in patients with multiple sclerosis from first clinical presentation Click here for additional data file. Supplemental material, sj-pdf-3-msj-10.1177_13524585211003479 for Longitudinal analysis of T1w/T2w ratio in patients with multiple sclerosis from first clinical presentation by Graham Cooper, Claudia Chien, Hanna Zimmermann, Judith Bellmann-Strobl, Klemens Ruprecht, Joseph Kuchling, Susanna Asseyer, Alexander U Brandt, Michael Scheel, Carsten Finke and Friedemann Paul in Multiple Sclerosis Journal
