# Supplementary material for: Longitudinal analysis of T1w/T2w ratio in patients with multiple sclerosis from first clinical presentation
Source: Mult Scler. 2021 Apr 15;27(14):2180–90. doi: 10.1177/13524585211003479 (PMC8597181; doi:10.1177/13524585211003479)
Supplement: sj-pdf-1-msj-10.1177_13524585211003479 – Supplemental material for Longitudinal analysis of T1w/T2w ratio in patients with multiple sclerosis from first clinical presentation [file sj-pdf-1-msj-10.1177_13524585211003479.pdf]

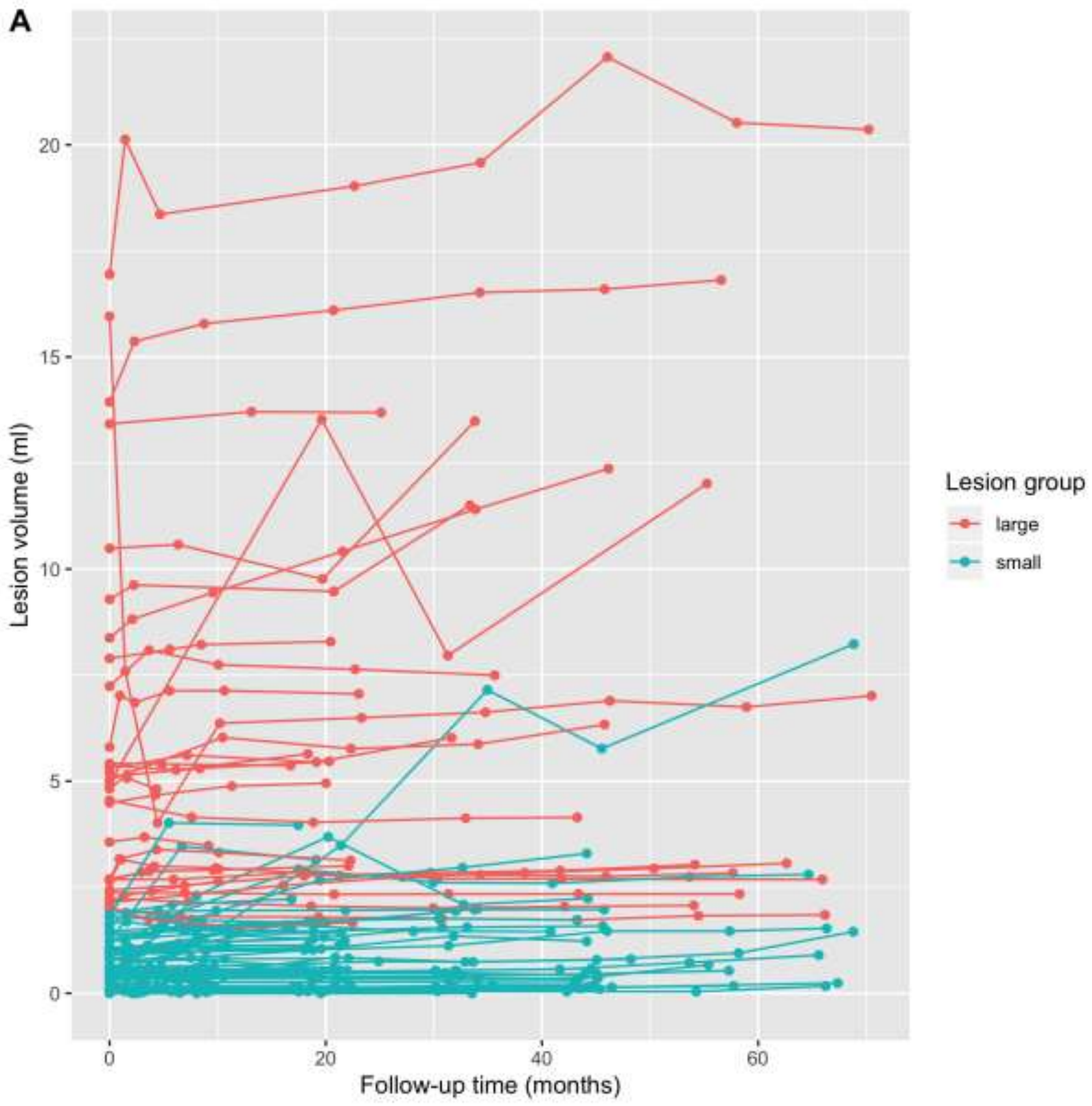

Supplementary Figure 1. **A)** Lesion volume (ml) over time for patients with a lesion volume > 2 ml at baseline (large) or < 2ml at baseline (small).

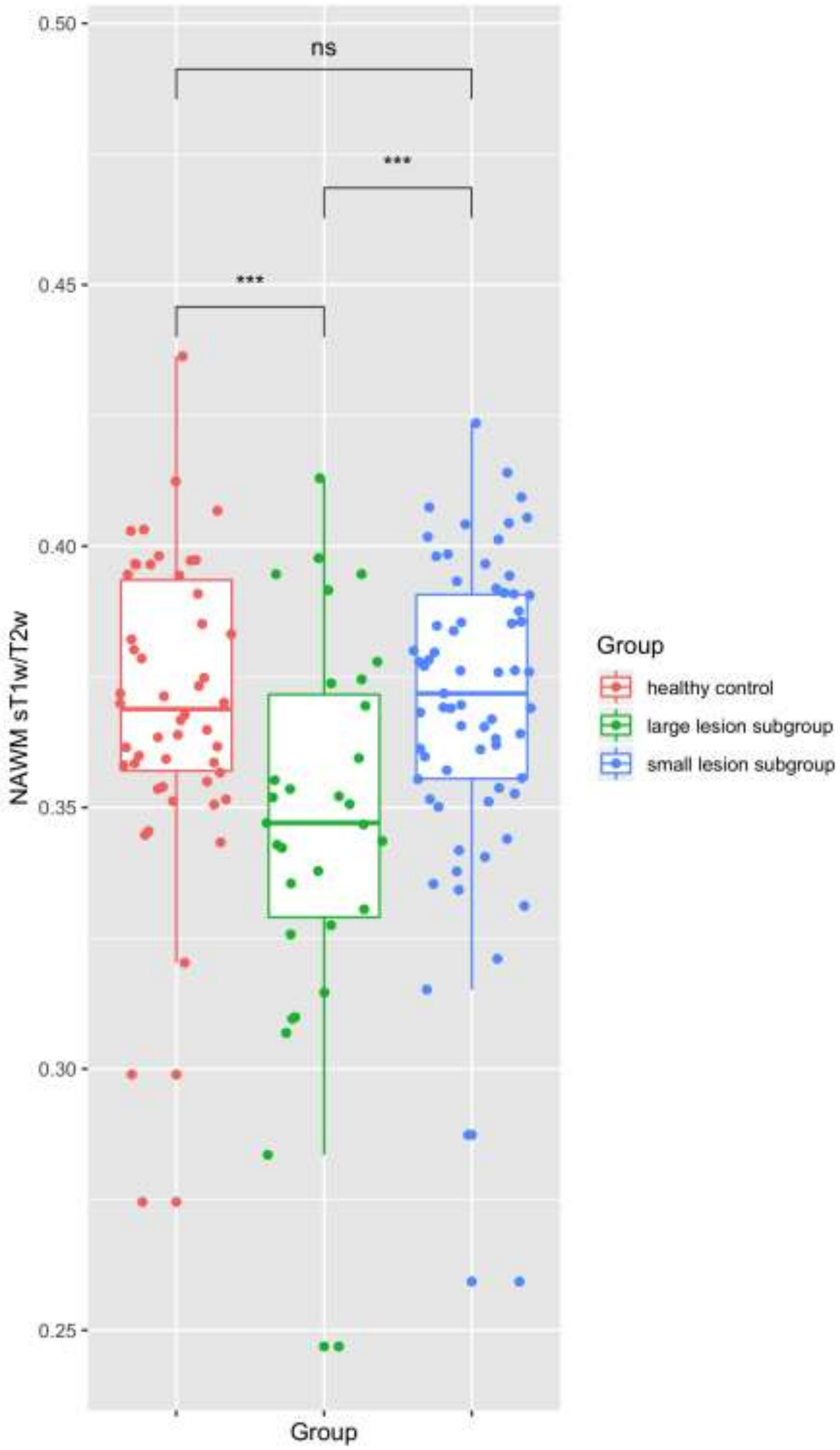

B) Comparison of baseline NAWM sT1w/T2w between healthy controls and lesion subgroups. ***p<0.001

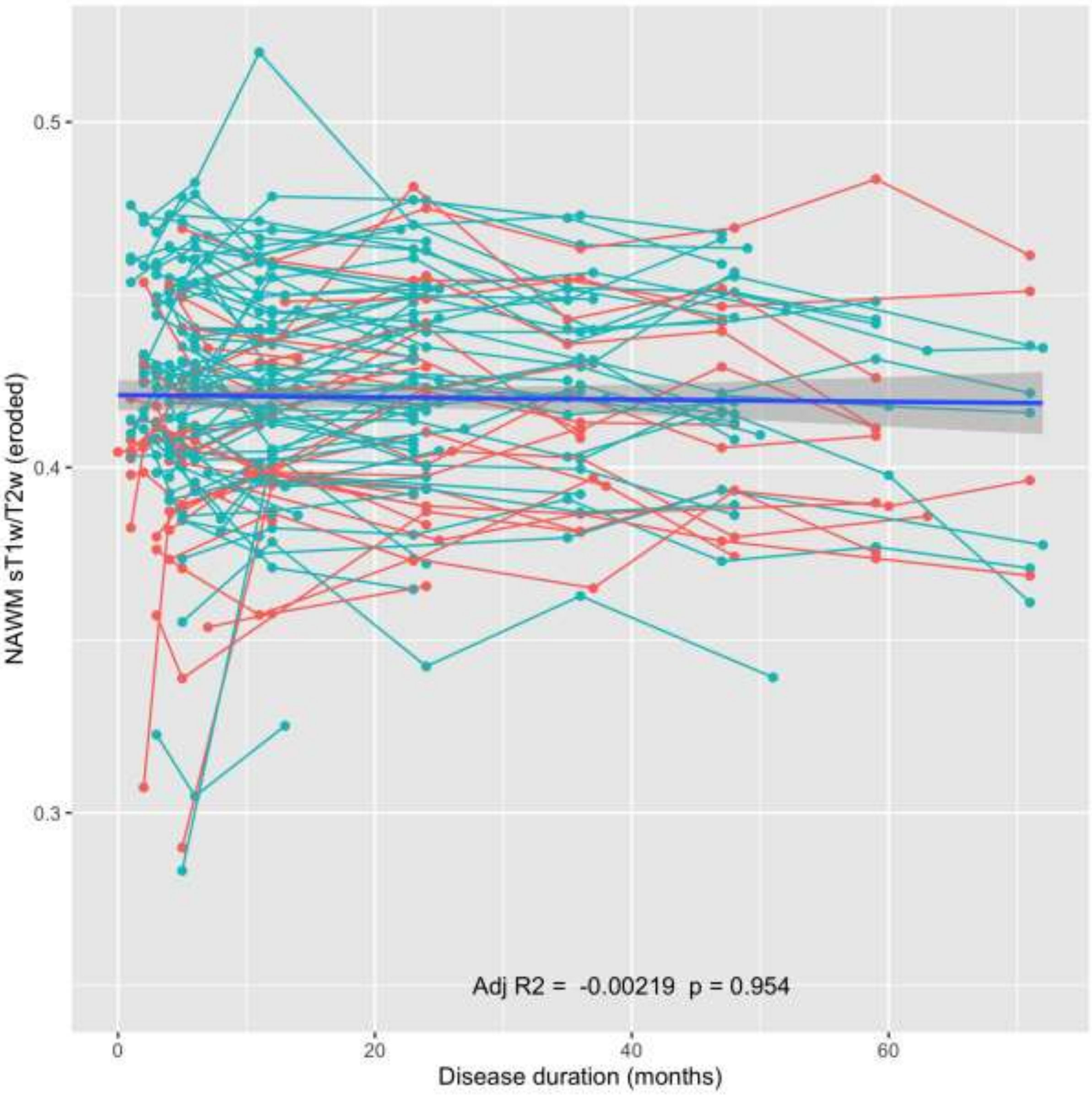

Supplementary Figure 2. Eroded NAWM sT1w/T2w ratio plotted against disease duration (months). Individual coloured lines show the trajectory of each parameter for individual patients. The thick blue line shows the regression line for the model parameter ~ disease duration for the whole cohort, including 95% confidence intervals (transparent blue). Adjusted R2 and p values are shown for this regression.

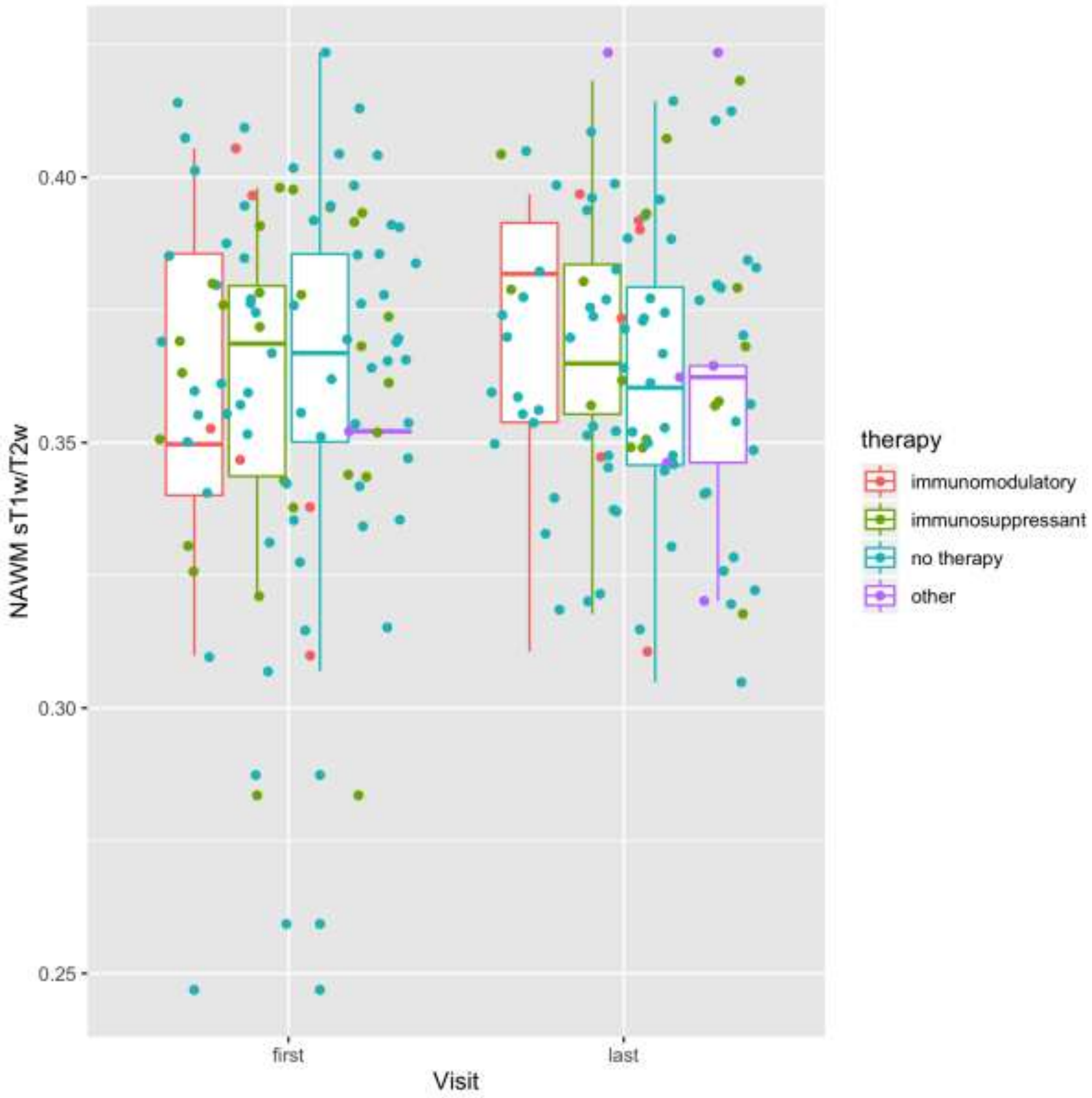

Supplementary Figure 3. NAWM sT1w/T2w compared between different therapies at baseline visit (first) and final visit (last).
