# Supplementary material for: Longitudinal analysis of T1w/T2w ratio in patients with multiple sclerosis from first clinical presentation
Source: Mult Scler. 2021 Apr 15;27(14):2180–90. doi: 10.1177/13524585211003479 (PMC8597181; doi:10.1177/13524585211003479)
Supplement: sj-pdf-2-msj-10.1177_13524585211003479 – Supplemental material for Longitudinal analysis of T1w/T2w ratio in patients with multiple sclerosis from first clinical presentation [file sj-pdf-2-msj-10.1177_13524585211003479.pdf]

Supplementary Data

Linear Mixed Model Formulae

Below we present the R formulae used to model all linear mixed models presented in the paper.

Association with Imaging Parameters

Model 1: linear mixed model of NAWM sT1w/T2w values with subject as a random effect and lesion volume interacting with time as conditional likelihood effect

lmer(NAWM sT1w/T2w ~ Baseline age + Follow up time*Lesion volume + (1 | subject))

Model 2: linear mixed model of NAWM sT1w/T2w values with subject as a random effect and cortical thickness interacting with time as conditional likelihood effect

lmer(NAWM sT1w/T2w ~ Baseline age + Follow up time*Cortical thickness + (1 | subject))

Model 3: linear mixed model of NAWM sT1w/T2w values with subject as a random effect and lesion volume and cortical thickness interacting with time as conditional likelihood effects

lmer(NAWM sT1w/T2w ~ Baseline age + Follow up time*Cortical thickness + Follow up time*Lesion volume + (1 | subject))

Association with NEDA-3

Model 1: linear mixed model of NAWM sT1w/T2w values with subject as a random effect and lesion volume and cortical thickness interacting with time and NEDA-3 as conditional likelihood effects

lmer(NAWM sT1w/T2w ~ Baseline age + Follow up time*Cortical thickness + Follow up time*Lesion volume + NEDA-3 + (1 | subject))

Model 2: linear mixed model of NAWM sT1w/T2w values with subject as a random effect and lesion volume, cortical thickness and NEDA-3 interacting with time as conditional likelihood effects

lmer(NAWM sT1w/T2w ~ Baseline age + Follow up time*Cortical thickness + Follow up time*Lesion volume + Follow up time*NEDA-3 + (1 | subject))

Model 3: linear mixed model of NAWM sT1w/T2w values with subject as a random effect and lesion volume and cortical thickness interacting with time as conditional likelihood effects and NEDA-3 as a random slope

lmer(NAWM sT1w/T2w ~ Baseline age + Follow up time*Cortical thickness + Follow up time*Lesion volume + (1 + NEDA-3 | subject))

Subgroup Analysis

Although on the group level NAWM sT1w/T2w did not significantly differ between patients and controls, the linear mixed model analysis showed an influence of disease activity, lesion volume and cortical thinning on NAWM sT1w/T2w. Therefore, we performed further sensitivity analyses. First, we investigated the difference between CIS and RRMS at baseline. CIS and RRMS significantly differed in lesion volume ($t = -6.3398$, $p < 0.001$) but not in disease duration ($t = -1.171$, $p = 0.246$), cortical thickness ($t = 0.885$, $p = 0.380$), or sT1w/T2w ($t = -1.596$, $p = 0.117$). Given the previously reported association between lesion volume and sT1w/T2w and the observed longitudinal distribution of lesions (Figure 2c), we split the cohort into two groups (Supplementary Figure 1a): patients with a total baseline lesion volume > 2 ml vs. < 2 ml. Patients in the large lesion volume group had significantly lower sT1w/T2w in NAWM compared to patients with smaller lesion volumes (Supplementary Figure 1b, Supplementary Table 1) and in comparison with healthy controls (Supplementary Figure 1b). However, these patients did not differ in any other imaging or clinical outcome (Table 4).

--- Supplementary Figure 1a here ---

--- Supplementary Figure 1b here ---

--- Supplementary Table 1 here ---

Evaluation of Partial Volume Effects on NAWM sT1w/T2w

Given that the standardization method is based on median gray matter intensities in T1w and T2w, it is possible that partial volume effects in segmentation could have a

systematic influence on the sT1w/T2w. In order to address this concern, we repeated the sT1w/T2w ratio using eroded gray and white matter masks, so that voxels with partial volume effects are removed.

Calculation of Eroded sT1w/T2w

First, the normal-appearing white and gray matter tissue masks were eroded by 1 voxel using fslmaths erode function. An eroded scaling factor was then calculated by dividing the median normal-appearing eroded gray matter intensity of the T1w image by the median normal-appearing eroded gray matter intensity of the T2w image. The T2w image was then multiplied by this eroded scaling factor to create an eroded scaled T2w image. Eroded sT1w/T2w was then calculated as described in the main paper, replacing scaledT2w with eroded scaled T2w in the equation.

Results using Eroded sT1w/T2w

Eroded NAWM sT1w/T2w did not differ between early MS/CIS patients and controls (0.41 vs 0.42, p = 0.604). Similarly, eroded NAWM sT1w/T2w did not significantly correlate with disease duration (Supplementary Figure 2; adjusted R^2 = -0.002, p = 0.954).

--- Supplementary Figure 2 here ---

The association between the eroded sT1w/T2w and other imaging parameters over time was assessed by replacing the NAWM sT1w/T2w values in model 3 with the eroded NAWM sT1w/T2w values. The results of the model were consistent with that of the main analysis (Supplementary Table 2). The association with NEDA-3 was similarly

repeated using the eroded NAWM sT1w/T2w and the results were also consistent with the main analysis (Supplementary Table 3).

--- Supplementary Table 2 here ---

--- Supplementary Table 3 here ---

The eroded NAWM sT1w/T2w was also significantly lower in patients in the large lesion subgroup compared to the small lesion subgroup (p = 0.012).

Evaluation of Longitudinal Gray Matter Pathology Effects on Scaling Factor

Because sT1w/T2w is standardized based on gray matter intensity values, it is possible that slight changes in the gray matter over time may systematically influence the scaling factor. This would result in sT1w/T2w reflecting both NAWM and gray matter pathology, rather than only NAWM. Therefore, we first investigated whether the scaling factor (median gray matter T1w intensity divided by median gray matter T2w intensity) systematically differed over time and found a small positive correlation with disease duration (adjusted R^2 = 0.012, p = 0.014). Given this small positive effect, we then investigated what disease-related imaging factors affect the scaling factor over time using the following linear mixed models: 1) fixed effect of baseline age, cortical thickness and lesion volume with a random effect of NEDA-3 and 2) fixed effect of baseline age, cortical thickness and lesion volume without NEDA-3 as a random effect. Using ANOVA, neither model out-performed the other so the model without NEDA-3

was determined to be the better model as it was more parsimonious. This model showed a significant effect of age but not of any imaging factors. Therefore, in the current cohort, it is unlikely that differences in sT1w/T2w are driven by invisible gray matter pathology.

Evaluation of Potential Therapeutic Effects on NAWM sT1w/T2w

We also investigated whether therapy could influence NAWM sT1w/T2w over the observation period. As shown in Table 1, the number of patients on therapy at baseline and final visit was very low (33.3% and 26.5%, respectively) and the type of therapy was also heterogeneous. We were therefore unable to include this information in the statistical modelling. However, we additionally plotted NAWM sT1w/T2w at baseline and final visit, grouped by therapy and show that there are no differences (Supplementary Figure 3). Therefore, a therapeutic effect on NAWM sT1w/T2w in the early disease stage seems unlikely.

--- Supplementary Figure 3 here ---
