# Supplementary material for: Longitudinal analysis of T1w/T2w ratio in patients with multiple sclerosis from first clinical presentation
Source: Mult Scler. 2021 Apr 15;27(14):2180–90. doi: 10.1177/13524585211003479 (PMC8597181; doi:10.1177/13524585211003479)
Supplement: sj-pdf-3-msj-10.1177_13524585211003479 – Supplemental material for Longitudinal analysis of T1w/T2w ratio in patients with multiple sclerosis from first clinical presentation [file sj-pdf-3-msj-10.1177_13524585211003479.pdf]

	Large lesion volume subgroup	Small lesion volume subgroup	Test statistic	Significance (*p*-value)
N	32	70		
age (median, range) (years)	32.8 (20.7 - 49.7)	33.2 (20.1 - 56.2)	t = -0.544	0.588
sex = M (%)	11 (34%)	23 (32%)	χ^2 = 0	1
Disease duration (median, range) (months)	4 (0 - 13)	4 (1 - 8)	W = 1085.5	1
Diagnosis at baseline = CIS (%)	0 (0%)	28 (40%)	χ^2 = 15.692	< 0.0001
Diagnosis at final visit = CIS (%)	0 (0%)	16 (22.9%)	χ^2 = 7.033	0.008
Follow-up duration (median, range) (months)	33.57 (3.44 - 70.5)	21.99 (5.69 - 68.8)	W = 1332	0.097
Baseline EDSS (median, range)	1.5 (0 - 3)	1.5 (0 - 3.5)	χ^2 = 7.527	0.275
Baseline T2 lesion volume (ml) (mean)	5.83 ± 4.22	0.67 ± 0.10	W = 2240	< 0.0001
Baseline Cortical thickness (mm) (mean)	2.61 ± 0.12	2.65 ± 0.10	t = -1.497	0.140
Baseline NAWM sT1w/T2w (mean)	0.35 ± 0.04	0.37 ± 0.03	t = -3.140	0.003
Fulfilled NEDA-3 criteria during follow-up (%)	5 (15.6%)	18 (25.7%)	χ^2 = 2.61	0.11

Supplementary Table 1. Demographic, imaging and clinical characteristics of the large lesion and small lesion subgroups.

	Model 3
age at baseline<br>coefficient (95% CI)	-0.001 ± 0.0004
follow-up time coefficient (95% CI)	0.0003 ± 0.001
lesion volume coefficient (95% CI)	-0.008 ± 0.003**
cortical thickness coefficient (95% CI)	0.021 ± 0.015*
Interaction between lesion volume and follow-up time coefficient (95% CI)	0.0001 ± 0.00005**
Interaction between cortical thickness and follow-up time coefficient (95% CI)	-0.0002 ± 0.0004
intercept (95% CI)	0.389 ± 0.045***
Number of patients	102
Number observations	455

marginal R^2	0.054
conditional R^2	0.834

Supplementary Table 2. Association between eroded sT1w/T2w and other imaging parameters. *p<0.1**p<0.05 ***p<0.01

	Model 3
age at baseline coefficient (95% CI)	-0.0005 ± 0.0004
follow-up time coefficient (95% CI)	-0.0004 ± 0.001
lesion volume coefficient (95% CI)	-0.010 ± 0.003***
cortical thickness coefficient (95% CI)	0.028 ± 0.014*
Interaction between lesion volume and follow-up time coefficient (95% CI)	0.0001 ± 0.00005

Interaction between cortical thickness and follow-up time coefficient (95% CI)	-0.0001 ± 0.0003
intercept (95% CI)	0.371 ± 0.043***
Number of patients	102
Number observations	356
marginal R^2	0.082
conditional R^2	0.896

Supplementary Table 3. Association between eroded sT1w/T2w, other imaging parameters and NEDA-3. *p<0.1**p<0.05 ***p<0.01
